# Identification of tophi in ultrasound imaging based on transfer learning and clinical practice

**DOI:** 10.1038/s41598-023-39508-5

**Published:** 2023-08-02

**Authors:** Tzu-Min Lin, Hsiang-Yen Lee, Ching-Kuei Chang, Ke-Hung Lin, Chi-Ching Chang, Bing-Fei Wu, Syu-Jyun Peng

**Affiliations:** 1 Division of Allergy, Immunology and Rheumatology, Department of Internal Medicine, School of Medicine, College of Medicine, Taipei Medical University, Taipei, Taiwan; 2 Division of Rheumatology, Immunology and Allergy, Department of Internal Medicine, Taipei Medical University Hospital, Taipei, Taiwan; 3 Institute of Electrical and Control Engineering, National Yang Ming Chiao Tung University, Hsinchu, Taiwan; 4 Professional Master Program in Artificial Intelligence in Medicine, College of Medicine, Taipei Medical University, No. 250, Wuxing St., Xinyi Dist., Taipei City, 110 Taiwan; 5 Clinical Big Data Research Center, Taipei Medical University Hospital, Taipei Medical University, Taipei, Taiwan

**Keywords:** Immunology, Rheumatology

## Abstract

Gout is a common metabolic disorder characterized by deposits of monosodium urate monohydrate crystals (tophi) in soft tissue, triggering intense and acute arthritis with intolerable pain as well as articular and periarticular inflammation. Tophi can also promote chronic inflammatory and erosive arthritis. 2015 ACR/EULAR Gout Classification criteria include clinical, laboratory, and imaging findings, where cases of gout are indicated by a threshold score of ≥ 8. Some imaging-related findings, such as a double contour sign in ultrasound, urate in dual-energy computed tomography, or radiographic gout-related erosion, generate a score of up to 4. Clearly, the diagnosis of gout is largely assisted by imaging findings; however, dual-energy computed tomography is expensive and exposes the patient to high levels of radiation. Although musculoskeletal ultrasound is non-invasive and inexpensive, the reliability of the results depends on expert experience. In the current study, we applied transfer learning to train a convolutional neural network for the identification of tophi in ultrasound images. The accuracy of predictions varied with the convolutional neural network model, as follows: InceptionV3 (0.871 ± 0.020), ResNet101 (0.913 ± 0.015), and VGG19 (0.918 ± 0.020). The sensitivity was as follows: InceptionV3 (0.507 ± 0.060), ResNet101 (0.680 ± 0.056), and VGG19 (0.747 ± 0.056). The precision was as follows: InceptionV3 (0.767 ± 0.091), ResNet101 (0.863 ± 0.098), and VGG19 (0.825 ± 0.062). Our results demonstrate that it is possible to retrain deep convolutional neural networks to identify the patterns of tophi in ultrasound images with a high degree of accuracy.

## Introduction

**Figure 1:**
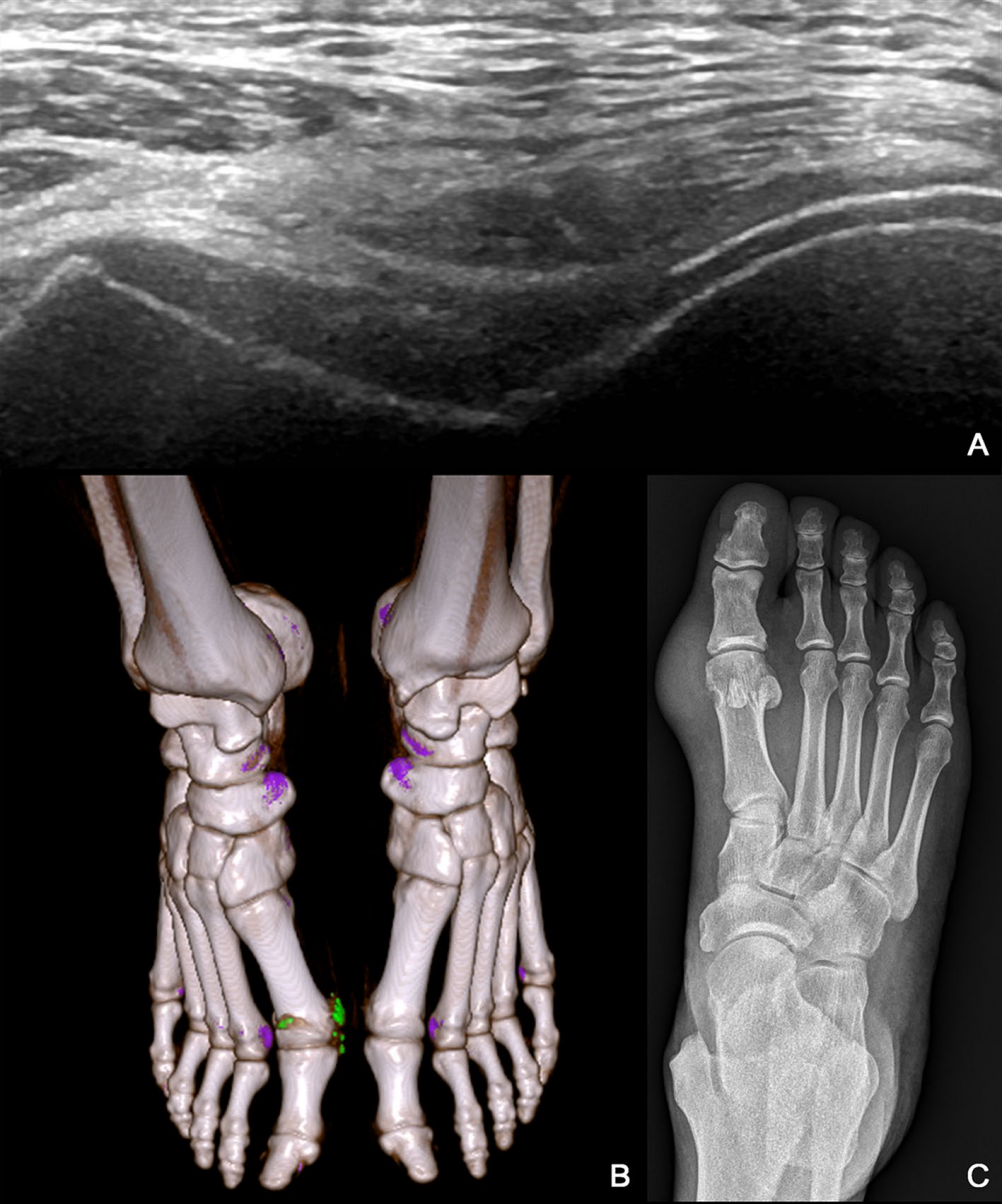
Imaging features included in the classification criteria: (**A**) Transverse ultrasonographic image showing double-contour in femoral articular cartilage. The image features hyperechoic enhancement over the surface of the hyaline cartilage; (**B**) Dual-energy computed tomography scan showing urate deposition at the right first metatarsophalangeal joint; (**C**) Conventional radiographic image showing signs of erosion in the first metatarsophalangeal joint, as indicated by a cortical break with sclerotic margin and overhanging edge.

Gout is a common metabolic disorder characterized by chronic hyperuricemia, i.e., the deposition of monosodium urate monohydrate crystals (tophi) in soft tissue, triggering intense and acute arthritis with intolerable pain as well as articular and periarticular inflammation^1^. Note that the prevalence of gout (< 1–6.8%) and the incidence of gout (0.58–2.89 per 1000 person-years) tend to vary widely due to the population studied and methods employed^2^. Tophi deposits are generally found at the 1st metatarsophalangeal (MTP) joints; however, they are occasionally found in the ankles and knees. Tophi has also been shown to promote chronic inflammation and erosive arthritis^3^. The gold standard for the diagnosis of gout is arthrocentesis^4^; however, imaging is increasingly being used to confirm the diagnosis due to its noninvasiveness^5^. The American College of Rheumatology (ACR) and the European League Against Rheumatism (EULAR) have collaboratively approved classification criteria for gout. Gout classification criteria in ACR/EULAR 2015 include clinical, laboratory, and imaging findings (Table 1), where cases of gout are indicated by a threshold score of ≥ 8^6^. Note however that some imaging-related findings, such as a double contour (DC) sign in ultrasound (Fig. 1A), urate in dual-energy computed tomography (DECT) (Fig. 1B), or radiographic gout-related erosion (Fig. 1C), generate a score of up to 4. Clearly, the diagnosis of gout depends heavily on imaging findings. Imaging has also been recognized as a useful tool to assess the outcomes of urate-lowering therapy^7^. Thiele et al. reported a strong correlation between DC characteristics and serum uric acid levels^8^. The Outcome Measures in Rheumatology (OMERACT) Gout Working Group has provided guidance for researchers on the means by which to assess the effects of treatment via imaging^9^. Imaging modalities have demonstrated good efficacy in detecting changes in urate deposition, joint inflammation, and bone erosions^10^. A substantial reduction in the size of tophi in ultrasound images is associated with a lower probability of relapse after terminating gout prophylaxis. Ultrasound follow-up can also be useful for managing urate-lowering therapy and gout flare prophylaxis^11–15^. DECT is the most accurate diagnostic imaging test; however, the procedure is expensive and exposes the patient to high levels of radiation^16^. Although musculoskeletal ultrasound is relatively non-invasive and inexpensive^17^, the reliability depends on expert experience. Studies have demonstrated that ultrasound offers good diagnostic accuracy with high specificity and is likely beneficial in the diagnosis of gout^18^. Researchers are increasingly looking to artificial intelligence (AI) to facilitate the interpretation of ultrasound images for diagnostic applications^19^; however, there has been relatively little research on the application of AI to the identification of musculoskeletal phenomena in ultrasound images. Our review of the literature pertaining to this issue revealed studies on myositis^20^, dysplasia of the neonatal hip^21^, synovitis^22^, lumbar ultrasound image feature extraction^23^, and nerve identification^24,25^. In the current study, we sought to use AI to identify the indicators of gout with a focus on detecting tophi at the 1st MTP joints.

**Table 1:** Definition and special considerations for each domain included in 2015 Gout Classification criteria.

Domain†	Definitions and special considerations
1. Pattern of joint/bursa involvement during symptomatic episode(s) with categories defined as per the description of the distribution of joints involved	Distribution of joint involvement: (i)Joint(s) or bursa(e) other than ankle, midfoot, or first metatarsophalangeal (MTP) joint (or their involvement only as part of a polyarticular presentation) (ii)Ankle or midfoot joint(s) as monoarticular or part of an oligoarticular presentation without first MTP joint involvement (iii)MTP joint involvement as monoarticular or part of an oligoarticular presentation
2. Characteristics of symptomatic episode(s): No characteristics present 1 Characteristic present 2 Characteristics present 3 Characteristics present	Characteristics to consider: (i)Difficulty walking or inability to use the affected joint(s) during a symptomatic episode (patient-reported) (ii)Pain when applying pressure to the affected joint during a symptomatic episode (patient-reported) (iii)Erythema overlying affected joint during a symptomatic episode (patient-reported or physician-observed)
3. Time course of symptomatic episode(s): No typical episodes One typical episode Recurrent typical episodes	Typical symptomatic episode including > 2 of the following, irrespective of anti-inflammation treatment: (i)Time to maximal pain < 24 h (ii)Resolution of symptoms in < 14 days (iii)Complete resolution (to baseline level) between symptomatic episodes
4. Clinical evidence of tophus: Present Absent Locations: Joints, ears, olecranon bursae, finger pads, or tendons (e.g., Achilles)	Appearance: Draining or chalk-like subcutaneous nodule beneath transparent skin, often with overlying vascularity (Fig. 2)
5. Serum urate level without treatment: < 4 mg/dl (0.24 mol/liter) 4–6 mg/dl (0.24–0.36 mol/l) 6–8 mg/dl (0.36–0.48 mol/1) 8–10 mg/dl (0.48–0.60 mol/l) ≧10 mg/dl (2:0.60 mol/l)	Special considerations: Ideally, serum urate levels should be tested within 4 weeks from the start of an episode and the patient was not receiving urate-lowering therapy. If practicable, then retest under those conditions. If serum urate level is ec:10 mg/dl, then no need to retest
6. Synovial fluid analysis: MSU negative Not performed	Location: Symptomatic joint or bursa Special considerations: Assessment should be performed by a trained observer Note: MSU positive is a sufficient criterion
7. Imaging evidence of urate deposition: Absent or not performed Present (Ultrasound or DECT)	Modality: Ultrasound or DECT Appearance: Double-contour sign on ultrasound (Fig. 3A)‡ or urate deposition on DECT (Fig. 3B)§ Location: Symptomatic joint or bursa
8. Imaging evidence of gout-related joint damage: Absent or not performed Present	Modality: radiography Appearance of gout-related erosion: Cortical break with sclerotic margin and overhanging edge; excluding gull wing appearance (Fig. 3C) Location: radiograph of hands and/or feet; excluding distal interphalangeal joints

*Symptomatic (ever) refers to pain and/or swelling.

^†^Categories within each domain are hierarchical. If a subject fulfills more than one category, then the highest category should be selected.

^‡^A false-positive double-contour sign (artifact) may appear at the cartilage surface, but it should disappear with a change in probe insonation angle.

^§^Images should be acquired using a dual-energy computed tomography (DECT) scanner, with data acquired at 80 kV and 140 kV and analyzed using gout-specific software with a 2-material decomposition algorithm that color-codes urate (33). A positive scan result is defined as the presence of color-coded urate at articular or periarticular sites. Nailbed, submillimeter, skin, motion, beam hardening, and vascular artifacts should not be interpreted as DECT evidence of urate deposition.

## Materials and methods

### Subjects

This study enrolled patients who were diagnosed with gout in accordance with 2015 ACR/EULAR gout classification criteria during the period from December 5th, 2019, to May 28th, 2020. The study was approved by Taipei Medical University Hospital Institute Ethics Committee (N202204073). All methods were performed in accordance with the relevant guidelines and regulations.

### Ultrasound imaging protocol

All images were obtained using a LOGIQ E9 ultrasound machine and ML6-15 ultrasound scanner by a single rheumatologist with 7 years of experience. The scanner was placed on the dorsal and lateral sides of the bilateral 1st MTP joints. A total of four images from each patient were used for training and evaluation.

### Region of interest (ROI) cropping and definition

**Figure 2:**
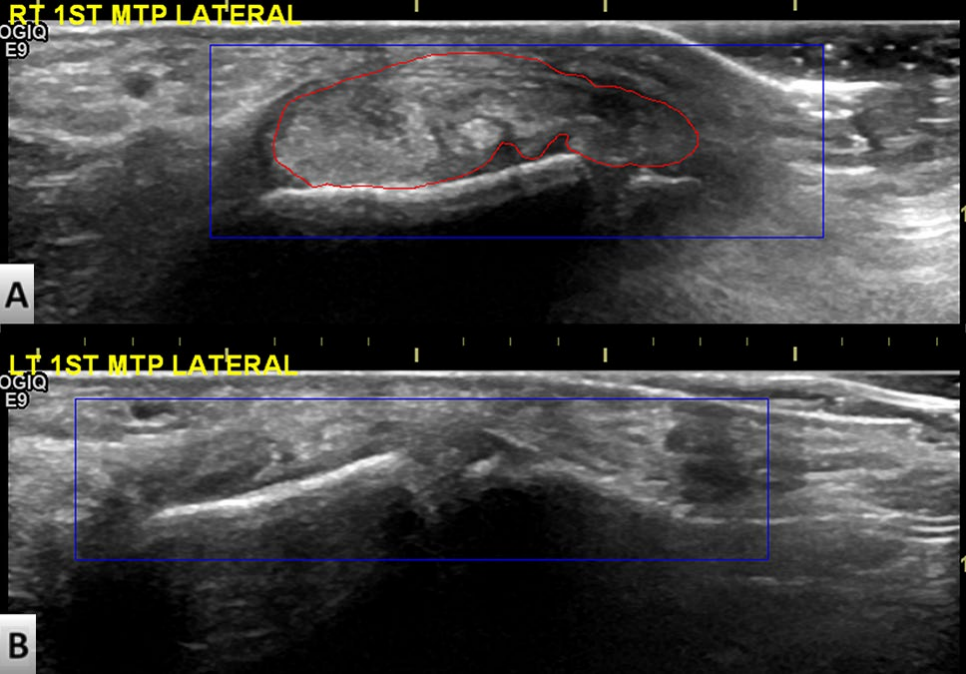
Ultrasound images of (**A**) left 1st metatarsophalangeal joint lateral side with tophi and (**B**) right 1st metatarsophalangeal joint lateral side without tophi in single gout patient. The red curve indicates the boundary of the tophi region. The blue rectangle frame indicates the region of interest drawn by a rheumatologist for subsequent transfer learning.

**Figure 3:**
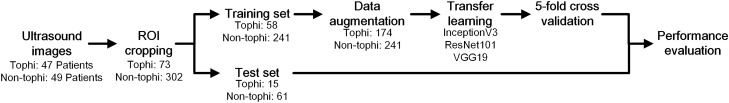
Schematic diagram showing the workflow of training and testing. After cropping the region of interest in 375 ultrasound images, the images were assigned to training or test datasets. Data augmentation based on histogram equalization and horizontal flipping was used to increase the number of images in the training dataset. Transfer learning was performed using three pre-trained models with fivefold cross-validation prior to the analysis of diagnostic performance using the test set.

The ROI in the images was identified using Microsoft Paint. All ROIs were labeled by the same rheumatologist who performed the scanning. Note that the size of the ROI was not stipulated. Rather, we focused on the bilateral 1st MTP joints and tophi. The blue square and the red label in Fig. 2 respectively indicate the ROI and tophi. The hyperechoic nature of tophi (and occasional calcification) generally results in a heterogenous appearance in scans^26^. Figure 3 presents a schematic diagram showing the workflow employed for training and testing. After ROI cropping, the images were split into a training and test set. Data augmentation based on histogram equalization and horizontal flipping was used to increase the number of images in the training set.

### Study design

The diagnoses of all tophi in this study were subject to confirmation by a rheumatologist, such that training was implemented as supervised learning. Transfer learning and the fine-tuning of hyperparameters were implemented on three pre-trained models: InceptionV3, ResNet101, and VGG19. Note that the classification accuracy of these models has been demonstrated in the ImageNet Large-Scale Visual Recognition Challenge. The MATLAB 2021a platform was used for the re-training of the three models specifically for the classification of tophi versus non-tophi in ultrasound images. The size of the input images was adjusted in accordance with model settings. Stochastic gradient descent with momentum was applied as the solver. The maximum number of epochs was as follows: InceptionV3 (20), ResNet101 (20), and VGG19 (30). The learning rate was as follows: InceptionV3 (0.0001), ResNet101 (0.0001), and VGG19 (0.0001). Five-fold cross-validation was used to ensure the stability of the results.

### Evaluation metrics

In estimating the diagnostic performance of physicians, the images were classified as tophi and non-tophi according to sonographic patterns. Model evaluation was based on several evaluation metrics, including accuracy, recall (sensitivity), precision (positive predictive value, PPV), F1-Score, receiver operating characteristic (ROC) curve, and area under the curve (AUC). The evaluation metrics were calculated based on the calculation of true negative (TN), true positive (TP), false negative (FN), and false positive (FP). TP and TN were respectively defined as the number of positive and negative cases that were successfully classified. FP and FN were respectively defined as the number of misclassified negative and positive cases. Accuracy was calculated as the ratio of correct predicted classes to the total number of samples evaluated. Recall was used to compute the fraction of positive patterns that were correctly classified. Precision was used to compute the positive patterns that were correctly predicted based on all predicted patterns in a positive class. F1-score indicated the harmonic average between recall and precision rates.

## Results

### Study population

Our initial recruitment included 111 patients. From this group, 15 patients were excluded due to unclear imaging data. This left 96 patients who met the enrollment criteria for this study, including those with tophi (*n* = 47) and those without tophi (*n* = 49). The initial plan was to use 4 images from each patient, for a total of 384 images. However, the exclusion of 9 images due to a lack of clarity resulted in 375 images, including 73 images with tophi and 302 images without tophi.

### Demographics

The patients were divided into a tophi group and a non-tophi group. Patient ages ranged from 22 to 87 years old. The mean age of patients was 52 years old (male: 52 years old, female: 64.5 years old). The ratio of male: female was 92:4. The mean uric acid level was 7.04 mg/dl (male: 7.08 mg/dl, female: 6.1 mg/dl).

### Performance assessment of AI models

**Figure 4:**
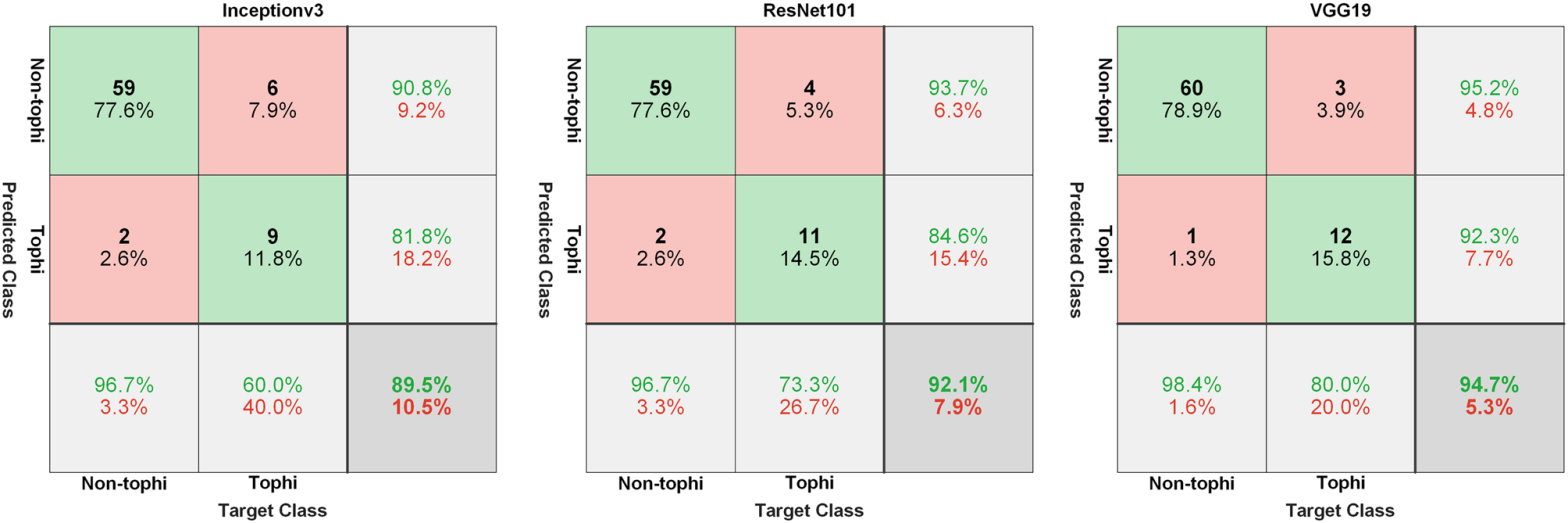
Confusion matrix of models when applied to the test set: (**a**) InceptionV3, (**b**) ResNet101, and (**c**) VGG19.

After data augmentation, the training set included 174 images in the tophi group and 241 in the non-tophi group. Following the completion of transfer learning, a test set was used to assess the performance of the models and obtain a confusion matrix (Fig. 4). Table 2 presents the accuracy of the models in terms of diagnostic performance, as follows: InceptionV3 (0.871 ± 0.020), ResNet101 (0.913 ± 0.015), and VGG19 (0.918 ± 0.020). Recall performance was as follows: InceptionV3 (0.507 ± 0.060), ResNet101 (0.680 ± 0.056), and VGG19 (0.747 ± 0.056). Precision was as follows: InceptionV3 (0.767 ± 0.091), ResNet101 (0.863 ± 0.098), and VGG19 (0.825 ± 0.062). AUC was as follows: InceptionV3 (0.925 ± 0.011), ResNet101 (0.966 ± 0.007), and VGG19 (0.967 ± 0.008).

**Table 2:** Performance evaluation of models in test dataset.

Test set	Accuracy	Recall	Precision	AUC
InceptionV3	0.871 ± 0.020	0.507 ± 0.060	0.767 ± 0.091	0.925 ± 0.011
ResNet101	0.913 ± 0.015	0.680 ± 0.056	0.863 ± 0.098	0.966 ± 0.007
VGG19	0.918 ± 0.020	0.747 ± 0.056	0.825 ± 0.062	0.967 ± 0.008

AUC, Area under the curve.

## Discussion

Neural networks comprise an input layer, a hidden layer for calculations, and an output layer to update the parameters of the hidden layer and train weights. The neurons in each layer are laid out within a fully connected structure. In 1988, Lecun et al. proposed the LeNet network architecture^27^, which laid the foundation for convolutional neural networks (CNNs). Their architecture included a convolution layer, activation function layer, pooling layer, flatten layer, and fully connected layer. A convolution kernel or filter slides across the image to extract features with the length and width of the image controlled by stride and padding. An activation function after the convolutional layer is used to derive a feature map via nonlinear transformation. A pooling layer is used to reduce the volume of image-related information by retaining only the information deemed important. The original information is then subjected to max pooling or mean pooling via a pooling filter, after which a feature map is created in the form of a 2D image. Finally, the feature map is expanded into a 1D array prior to insertion into the fully connected layer.

Currently dominating various computer vision tasks, CNNs are a class of artificial neural network that has been attracting immense interest across a variety of domains, including radiology. A CNN is designed to automatically learn and adapt spatial hierarchies of features through backpropagation using multiple building blocks, such as convolution layers, pooling layers, and fully connected layers^28^. CNNs are now widely utilized for the identification of anatomical structures in medical ultrasound image analysis^29^. In the rheumatology field, several studies have used CNNs for the measurement and estimation of metacarpal-head cartilage^30,31^. Visual observation and semi-quantitative analysis are commonly used in the interpretation of medical ultrasound images; however, visual analysis is subjective and reviewer-dependent while semi-quantitative methods are operator-dependent. Visual evaluation involves the careful examination of ultrasound images covering the ROIs to compare lesions with surrounding normal tissue. However, the results are easily affected by ambient lighting, screen brightness and contrast, eyestrain, and the clinical experience of the observer. Semi-quantitative methods involve converting the intensity of ROIs bound by radiotracers into a numeric value representing the characteristics of that specific lesion. Ideally, the results should be reproducible and strongly correlated with the results of visual analysis. Researchers have recommended the establishment of databases by which to derive observer-independent imaging protocols.

In patients with chronic gout, serum urate levels and the rate of urate deposition are primary outcome measures^32^. The gout working group at the OMERACT in 2014 reported that measuring whole-body urate deposition levels is not a feasible option. By contrast, it is possible to quantify tophi within a representative area (e.g., bilateral 1st MTP joints) or a predetermined set of joints^33^. Ultrasound and DECT are the methods best suited to measuring urate deposition^9^; however, DECT exposes patients to unacceptable levels of radiation and is available in few research centers. Ultrasound can also be used for the quantification of tophi deposition in superficial areas, such as the 1st MTP joints. It is inexpensive, non-intrusive, and widely available; however, the results are highly dependent on the specifics of the scanner and operator experience. Our objective in this study was to apply an AI learning system to the identification of tophi in ultrasound images. Our review of the literature revealed that this was the first-ever study to use transfer learning in the development of an AI system for the interpretation of ultrasound images aimed at identifying tophi in musculoskeletal tissue.

It is important to note that the properties of ultrasound images vary with the operator, scanner, and patient^34^. Our study also has some limitations. All of the images used in the current study were captured by the same experienced rheumatologist using the same ultrasound machine and scanner. Although this helped to ensure consistent image quality, it limited the diversity of images in the database.

## Conclusions

In our study, we applied an AI learning system to the identification of tophi in ultrasound images. Our results demonstrate that it is possible to re-train deep convolutional neural networks to identify the patterns of tophi in ultrasound images with a high degree of accuracy.

## Data Availability

The datasets generated and/or analyzed during the current study are not publicly available but are available from the corresponding author on reasonable request. During the data evaluation process, all data were anonymized.
